# Supplementary figures and images for: Drosophila Zic family member odd-paired is needed for adult post-ecdysis maturation
Source: Open Biol. 2019 Dec 18;9(12):190245. doi: 10.1098/rsob.190245 (PMC6936260; doi:10.1098/rsob.190245)

**CCAP>GFP**

**opa-lacZ**

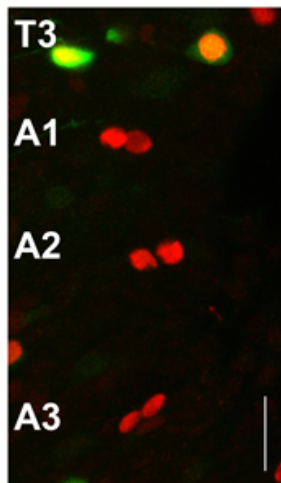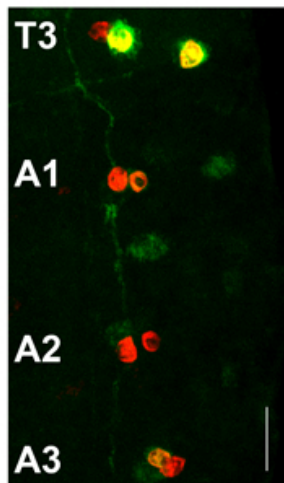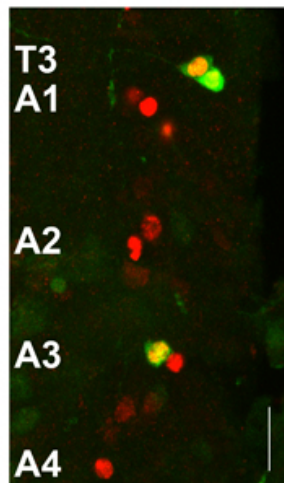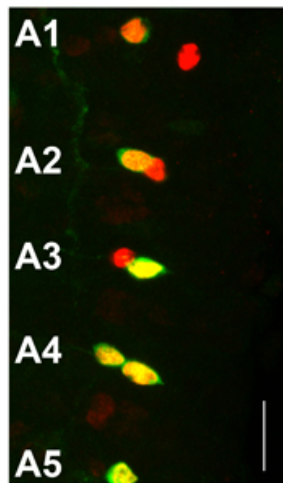

st. 12

----->

st. 17

Supplement: Figure S1 [file rsob190245supp1.pdf]

**opa-lacZ**

**odd-lacZ**

Control

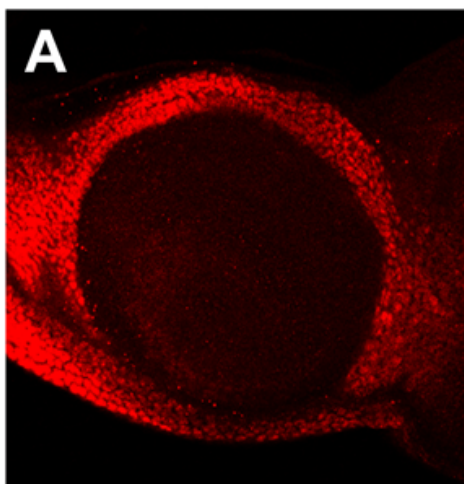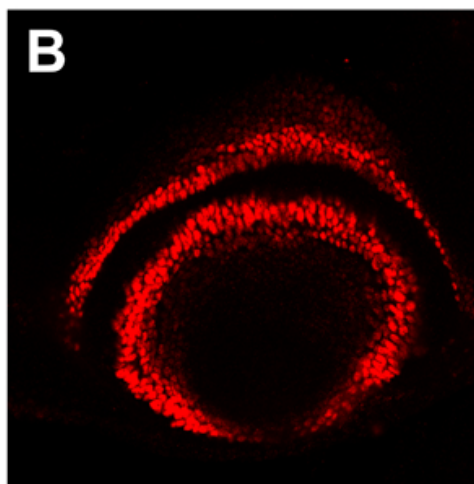

**GFP**

**odd-lacZ**

*FRT opa<sup>7</sup>*

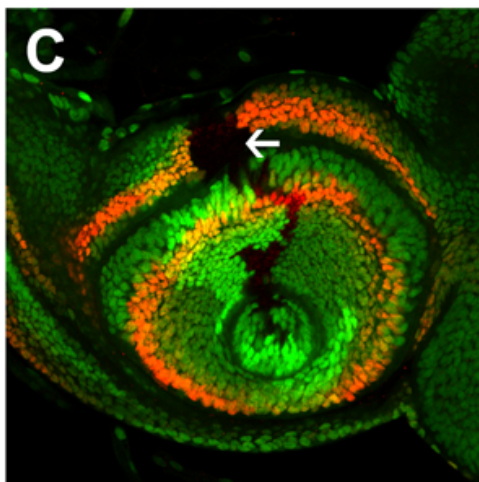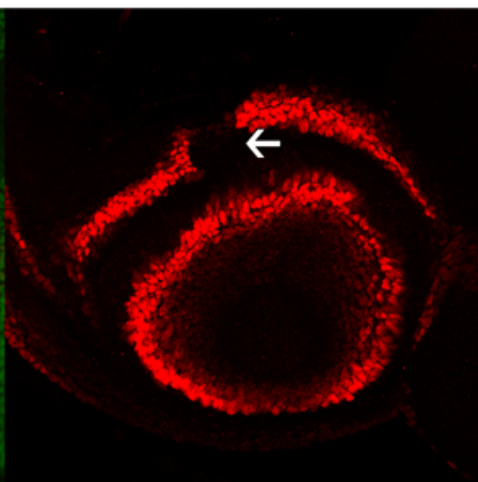

*tub>GFP>Gal4*  
*RNAi-opa<sup>II</sup>, UAS-Dcr-2*

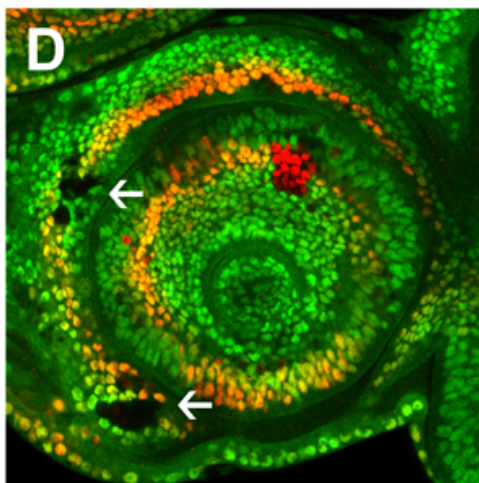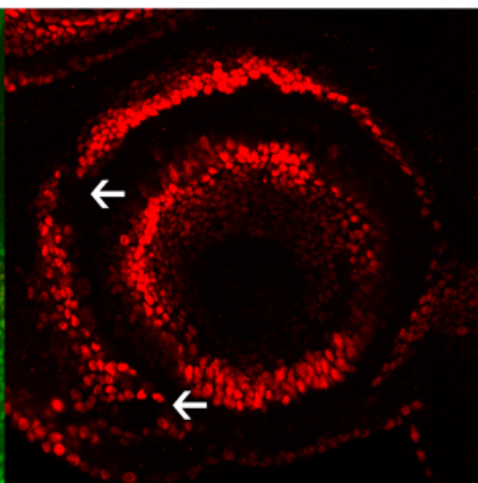

Supplement: Figure S2 [file rsob190245supp2.pdf]
